# From contact to connection: a comprehensive examination of affective touch in educational settings

**DOI:** 10.3389/fpsyg.2024.1230796

**Published:** 2024-04-24

**Authors:** Sonia El Hakim, Danyal Farsani

**Affiliations:** ^1^University of Valencia, Valencian Community, Valencia, Spain; ^2^Norwegian University of Science and Technology, Trondheim, Norway

**Keywords:** touch, teacher education, embodied cognition, engagement, embodiment

## Abstract

We often talk about the way we talk, and we frequently try to see the way we see, but for some reasons we have rarely touched on the way we touch. The communication we transmit with touch is perceived to be one of the most powerful means of establishing human relationships. In particular, tactile communication with parents, caregivers and teachers is particularly important for infants and students, as it helps make stronger relationships between educators or teachers and schoolers and also between students. Research has demonstrated the numerous benefits that an affective touch has on students, physically, socially and cognitively, or as has observed, touch touches deeper that just one’s skin and it is a recipe for creating meaningful relations. However, in the educational context, touch is perceived to be a complex phenomenon full of tension and emotion. For years, a dilemma has arisen in educational institutions in some countries, whether teachers can touch students or not? Despite the benefits that affective touch brings to students, cases of sexual abuse and inappropriate behavior at school have alerted the education system, to such an extent that many teachers worldwide consider what is and is not appropriate when communicating affectively with their students through touch. In this *perspective article*, by drawing on previous literature reviews, we shall highlight the benefits that affective touch has on learners.

## 1 Introduction

Educators’ bodies, embodiment, and touch are not just private, personal phenomena but socially shared and constructed (Merleau-Ponty, 1964). Merleau-Ponty observed that touch is a two-way road: One cannot touch without being touched at the same time (Merleau-Ponty, 1964), or as Jones and Yarborough (1985) have longed observed, one can not touch and, at the same time, be uninvolved with the other person. Touch has been studied in the context of schools (e.g., Heinonen et al., 2020) from the viewpoint of students (Keränen et al., 2020), educators (Johansson et al., 2021) and student teachers (Johansson et al., 2021). Touch, being an extension of proxemics (distance between people), is mediated by the culture of belonging (Hall, 1963; Hall, 1966; Watson, 1968) even in educational settings (Farsani, and Mendes, 2021; Farsani et al., 2022). Thus, the US, the United Kingdom or northern European countries are considered low-contact countries, while Arab, Mediterranean and Latin countries are considered high-contact cultures. This leads us to a reflection: most research on the positive effects of touch is carried out in low-contact countries. And, despite this, the results leave no room for doubt: positive interpersonal touch generates positive reactions and internal states in touch receptors, both in adults and children (Gallace and Spence, 2010; Field, 2019; Suvilehto et al., 2023). The notion of touch in schools is becoming more and more regulated in many countries, to the point we consider the possibility of achieving a scenario of a “zero contact” school, both in low and high contact countries. As Keränen and Uitto (2023, p.181) say, there is a big contradiction in educator’s work: “touch is simultaneously something to value and something to avoid.” At that point, this perspective article aims is to argue about this question: will the benefits of knowing that our students are not at risk of being touched inappropriately by their teachers outweigh the negative effects that deprivation of emotional touch has on them?

## 2 New perspectives

Ashley Montagu (1971) echoes a 1915 report by Dr. Henry Dwight Chapin, a distinguished pediatrician, in which he stated that in all but one of the orphanages in 10 different cities in the United States, the mortality rate for children under two was 100%. Babies died of a disease called “marasmus,” which was a weakness, an atrophy that had consequences as serious as premature death. And this despite the fact that the children received the “essential care”: hygiene, food, shelter etc. The orphanage in which the babies did survive was distinguished from the others precisely by including affective touch in these “essential cares.” Children are observed to need, for their proper psychological development and well-being, the affective touch on the part of their caregivers (Field, 2001, 2019; Barnett, 2005; Bergnehr and Cekaite, 2018). In fact, a lower emotional tact of parents toward their children correlates with aggressive behaviors of these toward their parents (Prescott, 1990; Field, 1999). According to Carlson and Nelson (2006), children perform aggressive touch because even that type of touch is better than the absence of touch (Owen and Gillentine, 2011).

Attachment theory, formerly developed by Bowlby (1969) and Ainsworth (1963) correlates warm behaviors from parents to their children such as physical closeness and touch with the perception of children of their parents as “sensitive, reliable, available and supportive” (Beetz et al., 2011, p. 351). Affective touch from caregivers is perceived to be one of the most important warm behavior to develop secure attachment in infants (Anisfeld et al., 1990; Duhn, 2010). Furthermore, it is also perceived to be important for developing attachment security in adults couples (Jakubiak and Feeney, 2016). Interestingly enough, Beetz et al. (2011) concluded that insecurely attached children prefer touching a dog rather touching a friendly person: as they find their parents to be rejecting and unsupportive, they avoid closeness with them. Insecurely attached children were generally unable to use the presence of another unfamiliar person for social support and stress alleviation.

The point for this perspective article is that this attachment is transferred to other close relationships like the one between children and their teachers (Bretherton, 1992). That means the kind of attachment a child has with her/his teachers is congruent with the attachment s/he has with his caregivers (Beetz et al., 2011). So, we can expect the kind of attachment of children with their parents will be determinant in the pleasantness experience of the touch from a teacher: securely attached children will receive touch from their teachers better than insecurely attached children.

Research has also provided evidence for the communication of emotions such as love, gratitude, and sympathy via touch (Hertenstein et al., 2006). Human touch is perceived to be necessary for life (Honig, 2005). Affective touch involves complex neurobiological processes such as release of oxytocin and endorphins, which contribute to generate calm and wellbeing state, the stimulation of special skin receptors (C-LTMRs, that is: C low threshold mechanoreceptors), which information is transmitted through the spinal cord and then reaches the brain through bottom-up pathways (Schirmer and McGlone, 2022). That way, affective touch appears to relieve physical pain, has numerous health benefits, such as improving the immune system, improving asthma, promoting sleep, physical growth (Field, 2001; Owen and Gillentine, 2011). Hugs are yet another form of touch which are perceived to reduce blood pressure and protect against increased heart rate in stressful situations (Grewen et al., 2003) and protect us from the common cold (Cohen et al., 2015). A friendly touch on the back or preschool children by an adult is perceived to improve their self-regulation to postpone gratification affecting not only their agreement to act as requested, but their decision-making and their will (Leonard et al., 2014).

The touching behavior can be understood in different ways depending on different variables (Burgoon et al., 1992; Ellingsen et al., 2016). Recipients’ touch perception depends on different internal elements like attention, internal motivational state, predictions of the meaning of touch, previous experiences (Ellingsen et al., 2016), gender (Stier and Hall, 1984; Hall and Veccia, 1990), age (Hertenstein et al., 2006), personality…, and external to the person touched such as culture (Hall, 1966; Dibiase and Gunnoe, 2004) and context (Macaluso and Driver, 2001), other nonverbal (Patterson et al., 1986; Burgoon, 1991; Burgoon et al., 1992; Soars, 2009; Ellingsen et al., 2014) and verbal (Bohm and Hendricks, 1997) cues or even interpersonal relationship between sender and recipient of touch. It also depends, of course on the physical characteristics of the touch, such as temperature, softness, force and velocity (Ellingsen et al., 2016).

This variety of determinants cause that in some cases, the hedonic experience of touch becomes unpleasant for the recipient (Ellingsen et al., 2016). However, the negative experiences in touch have been studied mainly in adults and in some clinic situations for children like autism (Riquelme et al., 2016).

In educational research, touch is often perceived as a natural and integral part of educators’ work, especially in early childhood education where young children are taken care of and nurtured via touch (Cekaite and Bergnehr, 2018; Svinth, 2018). In early childhood education, it is primarily though touch that educators can, for example, control students by setting rules such as “do not run,” or “sit still” (Lupton, 2013), using touch in its instrumental function (Burgoon et al., 1992; Rosa et al., 2020; Rosa and Farsani, 2021). The diverse functions of touch in care for students have been emphasized, such as controlling, compassionate, comforting, and affectionate touch (Bergnehr and Cekaite, 2018; Cekaite and Bergnehr, 2018). Affective touch appears to bring a physical, psychological and emotional benefit to students. The positive effects of touch in the educational environment have also been studied with university students, improving their conformity with the requests of their teacher (Guéguen, 2004; Leonard et al., 2014), but the positive effects on the attention of students aged five to six years have also been studied, as well as the reduction of disruptive behaviors when receiving positive touch by the teacher (Wheldall et al., 1986). Khatin-Zadeh et al. (2022, 2023) showed that it is through the medium of touch, gestures and embodiment that empower teachers to convey difficult and abstract mathematical concepts into a more tangible and transparent understanding. In another study (Owen and Gillentine, 2011) a survey from 63 teachers in the US was carried out and the results showed that 98% of teachers considered that touching children promotes their emotional development and 92% thought it reduced stress. However, only 30% of those same teachers touched children in situations of emotional discouragement, due to the fear that teachers have developed of being accused of abuse (Owen and Gillentine, 2011).

Previous research has illustrated tensions to exist between practices of touch and no-touch in educators’ work: On one hand, educators see touch as a natural touch way to be with students (e.g., Keränen and Uitto, 2023). On the contrary, touch is also perceived to be a risky behavior in education, since educators’ touch can be misinterpreted for example as a physical or sexual assault by students (Piper and Smith, 2003; Andrzejewski and Davis, 2008). Cases of sexual abuse in the classroom are very rare in the United States, but that has not prevented teachers from feeling afraid to touch their students, either a touch to impose discipline or an affective touch, for fear of being denounced (Owen and Gillentine, 2011). Putting the case of Spain, according to a 2021 report by the NGO defending children’s rights Save The Children, in which 394 judicial sentences on child sexual abuse were analyzed, it was found that 6% of these cases are related to educators, compared to 49.5% in which the aggressor is a relative. 9.7% where the aggressor is a partner or friend of the victim or 8.6% where the aggressor is an acquaintance of the family. Therefore, despite the fact that the cases in which the abuse of minors by teachers is the lowest percentage (far, of course, from a desirable 0%), it is only in this area that zero-contact campaigns by teachers toward their pupils have been promoted in some countries. In any case, we must be cautious with the figures of complaints in absolute values, since these may not reflect the reality of the abuses that actually occur in the classroom. And, when we talk about touch between adults, the meaning of tactile interaction with a sexual function is quite clear (Jones and Yarbrough, 1985). However, the function of touch performed by an adult on a child may not be understood by the child. This can lead to situations in which an inappropriate touch is not perceived by the child as such or, conversely, an “innocent” touch is misinterpreted by the child. We strongly believe more studies are needed to explore and better understand this phenomenon, particularly, from the perspective of the children and teacher educators.

## 3 Conclusion

This short *perspective article* aimed to raise awareness to the hidden messages of touch and how children consciously and/or unconsciously respond to it. Although this perspective appears to be ‘too one-sided’ on the positive evidence of touch, there could be fundamental reasons as to why it is the case. It could be anything ranging from the feeling of ‘shame’ or/and ‘embarrassment’ *talking about* (from the perspective of the children) or *reporting* (from the perspective of adults/researchers) the negative effects of touch. Although from one perspective this speculation is plausible, on the other hand, empirical studies have shown that teachers’ touch is observed to generate positive reactions and reduces disruptive behaviors in classrooms (e.g., Keränen and Uitto, 2023). Touch can be a mediatory tool for boosting students’ engagement in educational activities. Touch, if used properly, can enhance not only social relations but also “learning and teaching connections” (Rosa et al., 2020; Rosa and Farsani, 2021; Farsani and Villa-Ochoa, 2022); that is, it can strengthen students’ involvement in educational activities and create a context in which students are actively engaged in learning activities. Furthermore, we would like to stress and highlight that teachers’ touch appears to be a resource that can empower teachers and educators to better engage with their students. It seems that touch touches deeper than mere skin and touch needs to be studied further from the viewpoint of educators.

Furthermore, in our professional experiences that covers five countries (Iran, England, Chile, Norway and Spain), we have not found any teacher education courses that touches upon this multisided phenomenon and thus many newly graduated teachers may not be aware of the benefits of touch in their professional teaching practice. Touch appears to be a medium to create bonds between humans; but at the same time, it can serve as a tool to strengthen the connection between students’ minds and an educational setting. Again, we would like to stress and highlight that future teacher trainees must be able to understand that touch has/is a multisided phenomenon, and that touch in educators’ work is not just about setting rules of what is and what is not a proper touch, for example, where and when to touch, for how long, the duration and the angle of touch (approaching a student from their left hand side versus their right hand side). Furthermore, as contemporary researchers (Johansson et al., 2021; Keränen and Uitto, 2023) have observed, for touch to be tactful, it must be critically considered, discussed, and evaluated as part of teachers’ work in the teacher training courses. Finally, we strongly believe that such professional debated must have a place and a time where teachers can freely reflect and share their experiences about touch.

## Data availability statement

The original contributions presented in the study are included in the article/supplementary material, further inquiries can be directed to the corresponding author.

## Author contributions

SH and DF both worked on the idea and developed the idea together. All authors contributed to the article and approved the submitted version.
